# Gastric Volvulus as a Rare Sequelae of a Hiatal Hernia: A Case Report and Review of the Literature

**DOI:** 10.7759/cureus.104229

**Published:** 2026-02-25

**Authors:** George Ladas, Jake Wiepen, Christopher Stewart, Mitchell Fisher, Suporn Sukpraprut-Braaten, Charles Harper

**Affiliations:** 1 Medicine, Kansas City University of Medicine and Biosciences, Kansas City, USA; 2 Graduate Medical Education, Kansas City University, Kansas City, USA; 3 Graduate Medical Education, Kansas City University, Kansas City , USA; 4 Graduate Medical Education, Unity Health, Searcy, USA; 5 General Surgery, St. Mary's Medical Center, Blue Springs, USA

**Keywords:** acute abdomen, acute gastric ischemia, gastric volvulus, hiatal hernia, mesentero-axial gastric volvulus, paraesophageal hernia

## Abstract

Gastric volvulus is a rare but potentially life-threatening complication of a hiatal hernia that can result in gastric ischemia or necrosis if not promptly recognized and treated. A 51-year-old man presented with several hours of severe nausea, vomiting, and chest pain. Physical examination was notable for signs of acute distress, and the patient reported dark-colored emesis. Initial laboratory evaluation demonstrated leukocytosis and elevated serum lactate levels. Computed tomography of the chest and abdomen revealed a large hiatal hernia with herniation of the majority of the stomach into the thoracic cavity and findings concerning for mesoaxial gastric volvulus. Esophagogastroduodenoscopy demonstrated ischemic changes involving the gastric cardia. Given concern for gastric ischemia, the patient underwent urgent surgical intervention, including reduction of the hiatal hernia, cruroplasty reinforced with bio-synthetic mesh, and gastropexy to prevent re-torsion. The patient tolerated the procedure without intraoperative complications and demonstrated clinical improvement postoperatively, with resolution of symptoms and stabilization of laboratory abnormalities. This case highlights the importance of early recognition of gastric volvulus as a complication of a hiatal hernia and underscores the role of prompt imaging and surgical intervention to prevent gastric ischemia and necrosis.

## Introduction

Hiatal hernias are defined as herniation of the stomach through the esophageal hiatus into the mediastinum and are commonly encountered in Western populations [1]. Hiatal hernias are commonly encountered, particularly among older adults, with reported prevalence varying depending on the population studied [2,3].

Hiatal hernias are traditionally classified into four types. Type I (sliding) hernias, the most common variant, are characterized by cephalad displacement of the gastroesophageal junction into the thoracic cavity [1,3]. Type II (true paraesophageal) hernias involve herniation of the gastric fundus alongside a normally positioned gastroesophageal junction [3]. Type III hernias represent a combination of sliding and paraesophageal components, with both a portion of the stomach and the gastroesophageal junction herniated above the diaphragm [3,4]. Type IV hernias are defined by herniation of additional intra-abdominal organs, such as the colon or small bowel, into the thoracic cavity [3,4].

While many individuals with hiatal hernias are asymptomatic or experience mild gastroesophageal reflux, only a minority develop severe complications [4]. In rare cases, hiatal hernias may be complicated by gastric volvulus, defined as abnormal rotation of the stomach along its transverse (mesoaxial) or longitudinal (organoaxial) axis. Gastric volvulus can result in gastric outlet obstruction, ischemia, and potential necrosis, representing a surgical emergency. Acute gastric volvulus occurs in approximately 4% of hiatal hernia cases [5].

Here, we present the case of a 51-year-old male patient who required urgent surgical intervention for gastric volvulus arising from a hiatal hernia.

## Case presentation

**Figure 1:**
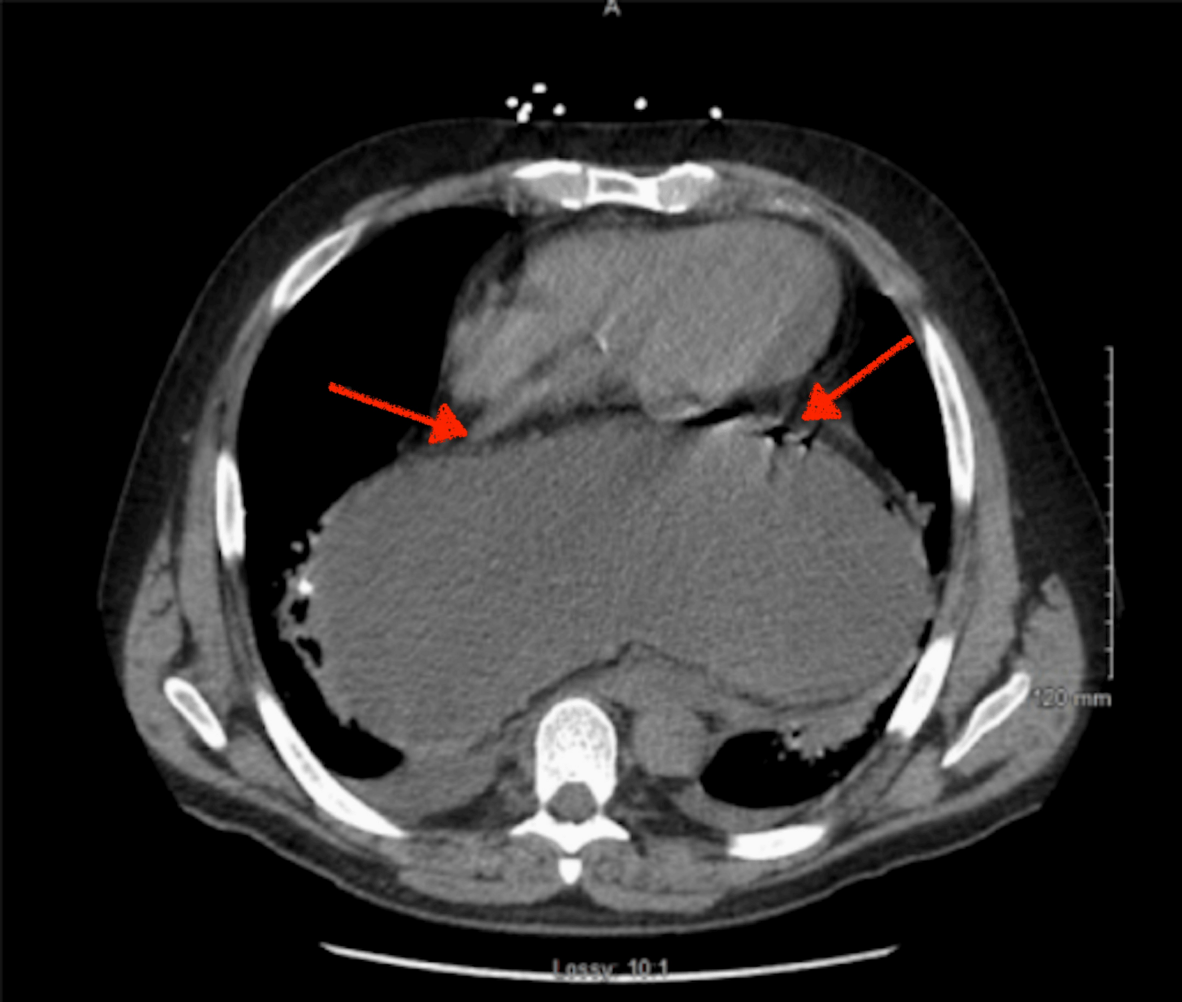
Axial computed tomography demonstrating intrathoracic displacement of the stomach (left arrow) with associated intramural gas within the gastric wall (right arrow). These findings are concerning for gastric ischemia in the setting of mesenteroaxial gastric volvulus and a large hiatal hernia.

**Figure 2:**
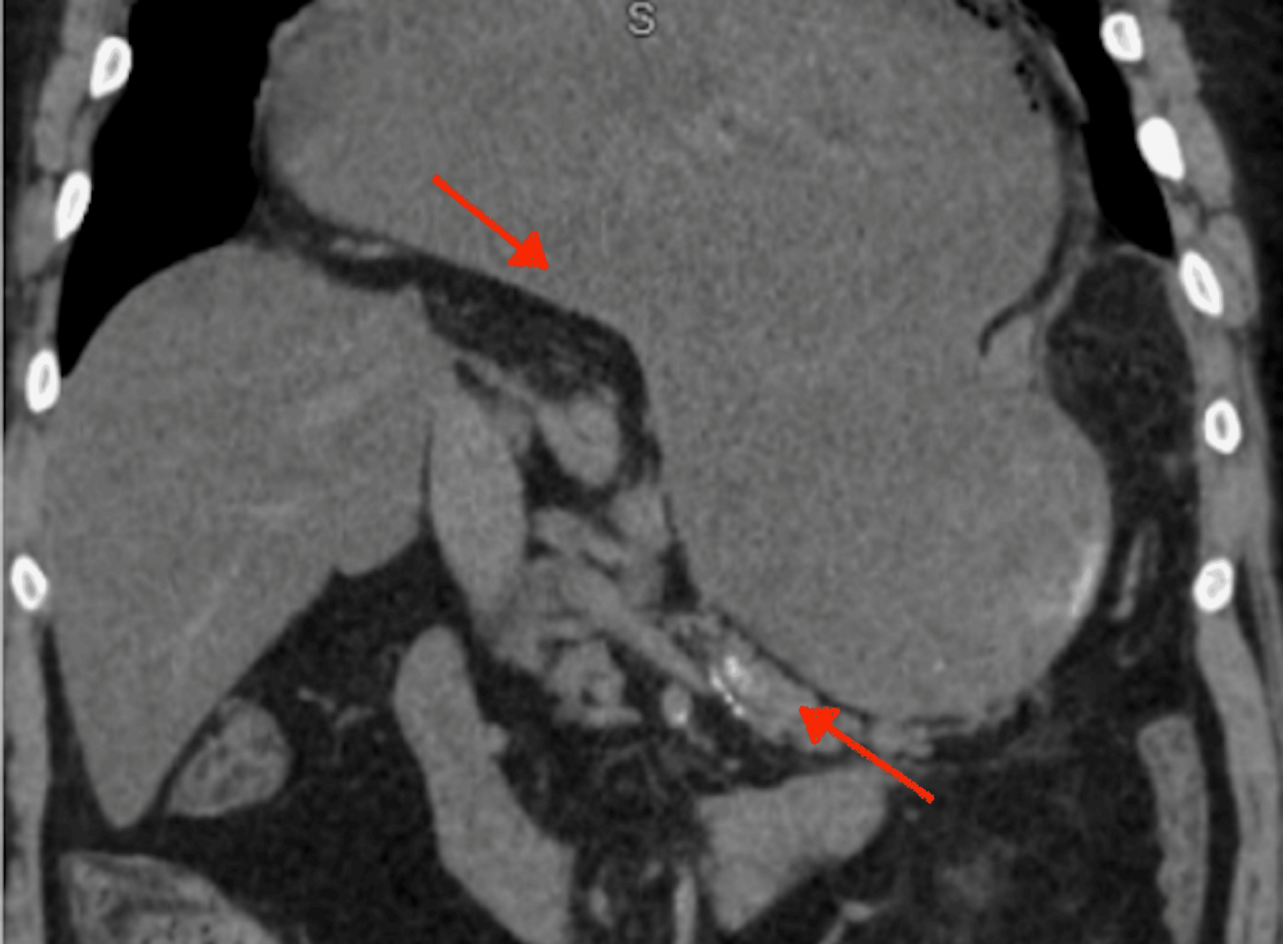
Coronal computed tomography demonstrating intrathoracic displacement of the stomach with a transition point reflecting abnormal rotation (arrows) These findings are consistent with mesenteroaxial gastric volvulus in the setting of a large hiatal hernia.

A 51-year-old man presented with several hours of intractable nausea, vomiting, and chest pain. Initial laboratory evaluation revealed leukocytosis and markedly elevated serum lactate levels (Table 1). The patient subsequently developed dark-appearing emesis. Computed tomography of the chest and abdomen demonstrated a large hiatal hernia with herniation of the majority of the stomach into the thoracic cavity, findings concerning for mesoaxial gastric volvulus, and areas of intramural gas within the gastric wall consistent with pneumogastrium (Figures 1, 2). Nasogastric decompression was performed, resulting in partial improvement of symptoms, and the patient was admitted to the intensive care unit for close monitoring and resuscitation. Gastroenterology was consulted, and esophagogastroduodenoscopy demonstrated ischemic changes involving the gastric cardia. Given concern for ongoing ischemia, urgent surgical intervention was undertaken. Intraoperatively, the hiatal hernia was reduced, and cruroplasty was performed and reinforced with bio-synthetic mesh. Persistent folding of the stomach at the prior site of torsion necessitated gastropexy to prevent re-torsion. The postoperative course was uncomplicated, and the patient was discharged on postoperative day three.

**Table 1:** Laboratory findings on presentation Laboratory values obtained at the time of initial presentation prior to surgical intervention.

Laboratory Test	Patient Value	Units	Reference Range
White blood cell count	20.1	×10³/μL	4.5–11.0 ×10³/μL
Serum lactate	8.1	mmol/L	0.5–1.0 mmol/L

## Discussion

Gastric volvulus typically develops when the stabilizing ligaments of the stomach become weakened or disrupted, allowing abnormal gastric mobility that predisposes the organ to rotation [3]. This loss of anatomic fixation is frequently associated with diaphragmatic defects such as hiatal or paraesophageal hernias, predisposing the stomach to abnormal rotation along either its longitudinal (organoaxial) or transverse (mesenteroaxial) axis [6]. Organoaxial volvulus involves rotation between the gastroesophageal junction and pylorus, whereas mesenteroaxial volvulus results in anterior and superior displacement of the gastric antrum [7].

Patients with acute gastric volvulus often present with severe epigastric pain, persistent retching, and inability to pass a nasogastric tube [8]. However, as demonstrated in this case, presentation may be variable, and the absence of the complete triad does not exclude the diagnosis. The patient exhibited acute nausea, vomiting, and chest pain but did not demonstrate all classic features, underscoring the diagnostic challenge and the importance of maintaining clinical suspicion in atypical presentations.

The most serious complication of gastric volvulus is vascular compromise resulting from torsion of the stomach and its associated blood supply. Progressive rotation can lead to strangulation, ischemia, and eventual gastric necrosis if left untreated [9]. Proximal gastric volvulus may impair perfusion to distal gastric segments, placing patients at risk for perforation and systemic instability.

Initial management focuses on hemodynamic stabilization and prompt diagnostic imaging, with computed tomography serving as the modality of choice for rapid identification of volvulus and associated complications [10]. Nasogastric decompression may provide symptomatic relief; however, definitive management depends on the presence of ischemia and patient stability. Endoscopic evaluation offers both diagnostic and therapeutic benefits by allowing direct visualization of the gastric mucosa and potential detorsion in select cases [4]. Evidence of ischemia, as seen in this patient, necessitates urgent surgical intervention.

Surgical management typically includes reduction of the herniated stomach, repair of the diaphragmatic defect via cruroplasty reinforced with bio-synthetic mesh, and gastropexy to prevent re-torsion. Fundoplication may be performed to reduce the risk of future herniation and reflux [2,3]. Prompt intervention is critical, as delayed recognition has been associated with poor outcomes, including perforation and mortality [7,10].

This case emphasizes the need for rapid clinical assessment and prompt intervention in patients with suspected gastric volvulus, particularly in the setting of a hiatal hernia. Notably, the patient presented with an atypical mesenteroaxial gastric volvulus without the complete Borchardt triad, underscoring the limitations of relying solely on classic diagnostic criteria. Despite initial symptomatic improvement with nasogastric decompression, endoscopic evaluation revealed ischemic changes that necessitated urgent operative intervention. This emphasizes the critical role of early endoscopic assessment in guiding management decisions and preventing ischemic complications, even when initial stabilization is achieved.

## Conclusions

Although hiatal hernias are often asymptomatic or associated with mild reflux, they may rarely progress to acute gastric volvulus with resultant ischemia. Clinicians should maintain strong diagnostic awareness in patients presenting with acute chest or upper gastrointestinal symptoms, particularly when laboratory abnormalities suggest evolving ischemia. Prompt diagnostic imaging and endoscopic evaluation are essential for identifying gastric ischemia and guiding timely surgical intervention.

## References

[1] Watson TJ, Ziegler KM (2022). The pathogenesis of hiatal hernia. Foregut.

[2] Dunn CP, Patel TA, Bildzukewicz NA, Henning JR, Lipham JC (2020). Which hiatal hernia’s need to be fixed? Large, small or none?. Ann Laparosc Endosc Surg.

[3] Smith RE, Shahjehan RD (2024). Hiatal hernia. StatPearls [Internet].

[4] Haro A, Abad L, Avila E (2023). Hiatal hernia: panoramic review of diagnosis and management. EPRA Int J Multidiscip Res.

[5] Masabarakiza JB, Zhu L, Gorur Y, Cardos B, Lorenzo-Villalba N, Ali D (2021). An unusual case of acute dyspnoea: acute intrathoracic gastric volvulus with probable tension gastrothorax. Eur J Case Rep Intern Med.

[6] Cribbs RK, Gow KW, Wulkan ML (2008). Gastric volvulus in infants and children. Pediatrics.

[7] Zafar M, Parvin J, Mcwhirter A (2022). Gastric volvulus: diagnosis and successful endoscopic de-rotation towards conservative management in a patient with multiple comorbidities. Cureus.

[8] Borchardt M (1904). On the pathology and treatment of gastric volvulus (Article in German). Arch Kiln Chir.

[9] Kalogeris T, Baines CP, Krenz M, Korthuis RJ (2024). Cell biology of ischemia/reperfusion injury. Int Rev Cell Mol Biol.

[10] Singham S, Sounness B (2009). Mesenteroaxial volvulus in an adult: time is of the essence in acute presentation. Biomed Imaging Interv J.

